# Evaluation of Single and Multi-Strain Probiotics with Gentamicin Against *E. coli* O157:H7: Insights from In Vitro and In Vivo Studies

**DOI:** 10.3390/microorganisms13020460

**Published:** 2025-02-19

**Authors:** Fatima H. Fneish, Khaled H. Abd El Galil, Souraya A. Domiati

**Affiliations:** 1 Department of Pharmaceutical Sciences, Faculty of Pharmacy, Beirut Arab University, Riad El Solh P.O. Box 11-5020, Beirut 11072809, Lebanon; 2 Department of Microbiology and Immunology, Faculty of Pharmacy, Mansoura University, Mansoura 35516, Egypt; kelgalil@hotmail.com; 3 Department of Pharmacology and Therapeutics, Faculty of Pharmacy, Beirut Arab University, Beirut 11072809, Lebanon; t.domyati@bau.edu.lb

**Keywords:** *E. coli* O157:H7, infection, gentamicin, probiotics

## Abstract

The emergence of antibiotic-resistant food-borne pathogens, especially *Escherichia coli* O157:H7, highlights the urgent need for innovative treatment strategies, particularly in light of rising resistances and the ongoing controversy surrounding antibiotic use in response to *E. coli* O157:H7 infections. To address this issue, we explored the potential of single- and multi-strain probiotics, both independently and in combination with gentamicin, through a series of in vitro and in vivo experiments. In vitro, gentamicin alone produced a mean inhibition zone of 12.9 ± 2.27 mm against *E. coli* O157:H7. The combination of gentamicin with single-strain probiotics (P1) increased the inhibition zone to 16.5 ± 2.24 mm (*p* < 0.05), while the combination with multi-strain probiotics (P2) resulted in the largest inhibition zone of 19 ± 2.8 mm (*p* < 0.05). In vivo, mice infected with *E. coli* O157:H7 and treated with P2, gentamicin (G), or their combination (G+P2), achieved 100% survival, no pathological symptoms, and full weight recovery within seven days. Conversely, mice treated with P1 or G+P1 exhibited lower survival rates (71.4% and 85%, respectively) and slower weight recovery. Hematological parameters improved across all groups, but kidney function analysis showed significantly higher serum creatinine levels in the P1, G, G+P1, and G+P2 groups compared to the P2 group (P1: 0.63 ± 0.15 mg/dL; G: 0.34 ± 0.09 mg/dL; G+P1: 0.53 ± 0.19 mg/dL; G+P2: 0.5 ± 0.23 mg/dL vs. P2: 0.24 ± 0.2 mg/dL). Histological analysis showed better intestinal and kidney tissue recovery in the P2 group, while the P1 and G+P1 groups exhibited abnormal ileal structures and severe cortical bleeding. These findings highlight the promise of multi-strain probiotics, alone or in conjunction with antibiotics, as a therapeutic strategy for *E. coli* O157:H7 infections. However, the nephrotoxicity associated with gentamicin co-administration remains a limitation, warranting further studies to optimize this approach.

## 1. Introduction

### 1.1. Background on E. coli O157:H7

Shiga-toxigenic *Escherichia coli* (STEC) serotype O157:H7 is a significant food-borne pathogen associated with several illnesses, including watery diarrhea, hemorrhagic colitis (HC), and hemolytic uremic syndrome (HUS) [1,2]. The bacterium was first identified as a cause of disease in 1982, following two outbreaks of bloody diarrhea in Oregon and Michigan, both linked to the consumption of hamburgers from a fast-food restaurant chain [3]. Since these early outbreaks, *E. coli* O157:H7 has emerged as one of the most significant foodborne pathogens worldwide, with numerous outbreaks reported globally [4,5,6,7,8]. The global incidence of *E. coli* O157:H7 infections is estimated at 2.8 million cases per year, highlighting its significant impact on public health [9].

### 1.2. Pathogenicity of E. coli O157:H7

The Pathogenicity of this bacterium is moderated through the release of Shiga-toxins (Stx1/or Stx2), which play a crucial role in the development of HUS [10]. Hemolytic uremic syndrome, a life-threatening complication, primarily affects children under 5 years of age and elderly patients over 60 years old [11]. It is characterized by a triad of symptoms: thrombocytopenia, acute renal impairment, and hemolytic anemia [12]. Ruminants and Cattle are the main reservoirs of STEC and the initial source of human infection [13]. Transmission typically occurs through the ingestion of contaminated food or water. In humans, STEC primarily targets the terminal ileum and colon, which serve as the main sites of colonization and disease [14].

### 1.3. Treatment Challenges

The current approach to treating *E. coli* O157:H7 infections is based primarily on supportive care, with antibiotic administration being a matter of debate. Some classes of antibiotics, such as quinolones, have been found to stimulate the release of Stx, leading to an increased risk of HUS development [15,16,17,18]. In contrast, many reports have shown that antibiotics like macrolides, carbapenems, and aminoglycosides may reduce Stx production, with gentamicin—an aminoglycoside—being extensively studied for its effects on *E. coli* O157:H7 [19,20,21,22,23]. However, due to these conflicting findings, the Infectious Diseases Society of America’s guidelines strongly recommends against the use of antibiotics in infections caused by STEC [24].

### 1.4. Potential of Probiotics in Managing Infections

As an alternative to antibiotics, probiotics have gained attention for their potential to treat STEC infection [25]. According to the World Health Organization (WHO), probiotics are defined as “live microorganisms that, when administered in adequate amounts, confer a health benefit on the host” [26]. Probiotics exhibit an antagonistic effect against pathogens by producing antimicrobial compounds such as bacteriocins. Additionally, they enhance the immune system and strengthen intestinal barrier function, improving the body’s overall defenses against infections [27,28]. The Food and Agriculture Organization (FAO) and the WHO have identified Lactic Acid Bacteria, *Bifidobacterium*, and the yeast *Saccharomyces boulardii* as the most commonly recognized probiotic microorganisms [29]. Evidence from several studies indicate that these probiotics may be beneficial in controlling *E. coli* O157:H7 infections [30,31,32,33,34,35]. Recent research has increasingly focused on the use of probiotics as adjuncts to antibiotics in various clinical settings. Studies have demonstrated that the combination of probiotics and antibiotics can yield improved outcomes in the treatment of many infections [36,37,38,39,40,41]. Reported benefits include a reduction in disease severity, reduction in antibiotic-associated side effects, increased eradication rates for certain infections, and a decrease in the development of antimicrobial resistance [42,43,44,45]. Despite these promising findings, the possibilities of this approach remain unexplored in the context of *Escherichia coli* O157:H7 infection.

### 1.5. Objective of the Study

The current study aimed to evaluate the effects of single- and multi-strain commercial probiotics on *E. coli* O157:H7, both alone and in combination with gentamicin, through in vitro and in vivo experiments. The study addresses the ongoing debate over gentamicin use and investigates the potential of probiotics as alternative treatment option. Moreover, by examining the combining effect of gentamicin and probiotics against *E. coli* O157:H7, the study seeks to gain a better understanding of this synergistic strategy, given the limited research available on its efficacy.

## 2. Materials and Methods

### 2.1. Collection and Molecular Screening of E. coli O157:H7

Between June and August 2020, a total of 250 clinical isolates of *E. coli* were collected from a hospital in the suburbs of Beirut. These isolates were evaluated for the presence of the *E. coli* O157:H7 serotype in the Microbiology laboratory at Beirut Arab University. A single colony of each isolate was transferred to Tellurite–Cefixime–Sorbitol MacConkey agar (TC-SMAC) and incubated at 37 °C for 24 h to evaluate the ability of the microorganism to ferment sorbitol. Non-fermenting sorbitol colonies were then subjected to molecular confirmation. Polymerase Chain Reaction (PCR) was conducted using specific primers for *E. coli* O157:H7, targeting the *rfbE* (forward primer: 5′-CATTGGCATCGTGTGGACAG-3′; reverse primer: 5′-AAGATTGCGCTGAAGCCTTTG-3′) and the *fliC* genes (forward primer: 5′-GCGCTGTCGAGTTCTATCGAGC-3′; reverse primer: 5′-CAACGGTGACTTTATCGCCATTCC-3′) [46,47]. These genes are responsible for encoding the somatic antigen O157 and the flagellar antigen, respectively. The PCR was prepared at a total volume of 20 μL, which included 5 μL of total DNA, 10 μL of 5× FIREPol master mix, 1 μL of each primer, and nuclease-free water to reach the final volume. The PCR cycling protocol began with a pre-denaturation step at 95 °C for 5 min, followed by 35 cycles: each cycle consisted of denaturation at 94 °C for 30 s, annealing at 60 °C for 30 s for the *rfbE* gene and at 68 °C for 30 s for the *fliC* gene, and extension at 72 °C for 50 s. A final extension step was performed at 72 °C for 5 min. The resulting amplification products were analyzed using agarose gel electrophoresis alongside a 100 bp DNA ladder (Invitrogen, Waltham, MA, USA).

### 2.2. Antimicrobial Susceptibility of the Isolates

The antimicrobial susceptibility of *E. coli* O157:H7 isolates was evaluated using the Kirby–Bauer diffusion method [48]. Fifteen antibiotics susceptibility disks (Oxoid Ltd., Basingstoke, Hampshire, UK) were used with the following variable concentrations: ampicillin (AMP, 10 μg), piperacillin (PRL, 100 μg) piperacillin/tazobactam (PRL, 100 μg), amoxicillin/clavulanic acid (AMC, 30 μg), ceftriaxone (CRO, 30 μg), ceftazidime (CAZ, 30 μg), cefepime (FEP, 30 μg) (5 μg), ciprofloxacin (CIP, 5 μg), norfloxacin (NOR, 10 μg), imipenem (IPM, 10 μg), meropenem (MEM, 10 μg), chloramphenicol (C, 30 μg), aztreonam (ATM, 30 μg), trimethoprim/sulfamethoxazole (SXT, 1.25/23.75 μg) and gentamicin (CN, 10 μg). For this purpose, *E. coli* O157:H7 colonies from a pure fresh culture were transferred to a tube containing 5 mL of Tryptic Soy Broth (TSB) and incubated at 37 °C for 24 h. The turbidity of the cultures was adjusted to match a 0.5 McFarland standard. Bacterial suspensions were then swabbed in three different directions on the surface of Muller–Hinton agar (MHA) plates and allowed to dry for 10 min. Antibiotic disks were placed on the MHA plates using an Oxoid antibiotic dispenser. The plates were left at room temperature for 15 min to enable pre-diffusion of the antibiotics, followed by incubation at 37 °C for 18–24 h. Zones of inhibition were measured with a Vernier caliper to the nearest millimeter. Results were categorized as susceptible (S), intermediate (I), or resistant (R) according to the inhibition zone diameter standards set by the Clinical and Laboratory Standards Institute [49].

### 2.3. Determination of MIC of Gentamicin

The minimum inhibitory concentrations (MICs) of gentamicin for *E. coli* O157:H7 isolates were assessed using the broth microdilution method following CLSI guidelines [50]. Sterile 96-well microtiter plates were used to establish a series of gentamicin concentrations, starting at 128 μg/mL and decreasing in two-fold dilutions down to 0.25 μg/mL. The bacterial inocula equivalent of the 0.5 McFarland standard were prepared from overnight cultures and subsequently diluted in nutrient broth at a ratio of 1:100. A volume of 100 μL of each inoculum was added to wells, resulting in a final concentration of 5 × 10^5^ CFU/mL.

For the negative controls, wells contained only nutrient broth to serve as a baseline for comparison. Positive wells included a mixture of 100 μL of bacterial inoculum and 100 μL of nutrient broth to confirm bacterial growth in the absence of antibiotics. The microtiter plates were sealed and incubated at 37 °C for 24 h. MIC values were determined as the lowest gentamicin concentration that completely inhibited bacterial growth. MIC interpretation breakpoints were established according to CLSI guidelines, with MICs ≤ 2 μg/mL classified as sensitive and MICs ≥ 8 μg/mL classified as resistant [51].

### 2.4. The Antimicrobial Activity of Commercial Probiotics Against E. coli O157:H7

Two types of commercial probiotics were purchased from a local pharmacy in Beirut. The composition of the single-strain probiotic (P1) and the multi-strain probiotic (P2) is presented in Table 1. Probiotic suspensions were prepared by rehydrating the contents of the capsules in normal saline to achieve a concentration of 10^8^ CFU/mL. The microorganisms present in the probiotics were confirmed by isolating each strain on appropriate selective media under specific incubation conditions, followed by identification using the VITEK 2 system. In this experiment, the agar well diffusion method was used to assess the efficacy of probiotics against *E. coli* O157 isolates as previously described [29,52]. Fresh bacterial cultures were prepared and standardized to a 0.5 McFarland standard before being evenly swabbed onto the surface of Mueller–Hinton agar (MHA). Using a cork borer, wells (8 mm in diameter) were created in the inoculated agar, into which probiotic suspensions were placed. After incubating the plates at 37 °C for 24 h, the presence of a clear zone around the wells was evaluated to determine the antibacterial activity of the tested probiotics.

### 2.5. The Antibacterial Effect of Probiotics in Combination with Gentamicin

To evaluate the combined antibacterial effect of probiotics and gentamicin, the methodology described by Moghadam et al. was followed. Fresh bacterial cultures of *E. coli* O157:H7 were standardized to a 0.5 McFarland standard. Bacterial inocula (25 μL) were swabbed evenly over the surface of solidified Mueller–Hinton agar (MHA) plates and allowed to dry for 10 min. A 20 μL volume of each probiotic suspension was applied to gentamicin disks, which were then left for 5 min to allow proper impregnation before being placed onto the surface of the agar plates. After a 24 h incubation period at 37 °C, the diameters of the resulting zones of inhibition were measured to assess the antibacterial efficacy of the combinations. The inhibition zones were recorded and analyzed to compare the effects of gentamicin alone versus its combination with single-strain and multi-strain probiotics [53].

### 2.6. In Vivo Evaluation of the Effects of Probiotics and Gentamicin on E. coli O157:H7

#### 2.6.1. Animals and Ethical Statement

Forty-nine male BALB/c mice, aged 6–8 weeks with an average weight of 23.4 ± 3.8 g, were obtained from the animal facility at Beirut Arab University and kept in the pharmacology department’s animal house. The mice were kept in standard cages under controlled conditions, including a 12 h light/dark cycle and a temperature of 24 °C. Throughout the experiment, they were provided with an autoclaved standard rodent diet and had free access to autoclaved water.

#### 2.6.2. *E. coli* O157: H7 Isolate

A gentamicin-sensitive isolate of *E. coli* O157:H7 was selected for the in vivo study. The bacterium was reactivated in Tryptic Soy Broth and incubated aerobically at 37 °C.

#### 2.6.3. Experimental Design

After a 48 h acclimatization period, the mice were randomly assigned to one of seven experimental groups, with seven mice in each group:

1. Control group: Not challenged with *E. coli* O157:H7.
2. Infected group: Challenged with *E. coli* O157:H7.
3. P1 group: Challenged with *E. coli* O157:H7 and administered the single-strain probiotic for 7 days post-infection.
4. P2 group: Challenged with *E. coli* O157:H7 and administered the multi-strain probiotic for 7 days post-infection.
5. G group: Challenged with *E. coli* O157:H7 and treated with gentamicin for 7 days post-infection.
6. G+P1: Challenged with *E. coli* O157:H7 and treated with a combination of the single-strain probiotic and gentamicin for 7 days post-infection.
7. G+P2: Challenged with *E. coli* O157:H7 and treated with a combination of the multi-strain probiotic and gentamicin for 7 days post-infection.

On day 1 of the experiment, following a 12 h fasting period, all groups, except the control group, received a single oral dose of 0.1 mL of *E. coli* O157:H7 at a concentration of 1 × 10^9^ CFU/mL, suspended in phosphate-buffered saline (PBS, pH = 7.3), as outlined in previous studies [54,55]. One hour after the bacterial inoculation, the P1 and P2 treatment groups were given 0.1 mL of probiotic suspensions, prepared by dissolving the contents of the capsules in normal saline to achieve a concentration of 10^8^ CFU/mL. Gentamicin was administered intraperitoneally based on the MIC value for the specific *E. coli* O157:H7 isolate used. The procedure lasted seven days, with each group receiving its designated treatment regimen daily. Mice were monitored each day, with their weight recorded and changes in body weight calculated based on the initial weight before inoculation. Clinical signs of illness, such as diarrhea, ruffled fur, lethargy, decreased activity, and bruising, were also observed and documented.

One-week post-infection, the mice were weighed and anesthetized with 80 mg/kg Ketamine and 8 mg/kg Xylazine by intraperitoneal injection for blood collection. Approximately 1 mL of blood was drawn through cardiac puncture and analyzed for complete blood count (CBC), blood urea nitrogen (BUN) and creatinine levels.

The kidney and colon tissues of the dead mice were promptly collected and fixed overnight in 10% formaldehyde. The tissues were then embedded in paraffin and stained with hematoxylin and eosin [56].

### 2.7. Statistical Analysis

Data were analyzed using SPSS software version 23 (IBM Corp, Armonk, NY, USA). Descriptive statistics, including means and standard deviations, were calculated to summarize the data. To assess the difference between groups, an ANOVA test was performed, followed by a post hoc test (Tukey’s test). A *p*-value of ≤0.05 was considered statistically significant with a confidence interval of 95%.

## 3. Results

### 3.1. Molecular Screening of E. coli O157:H7

**Figure 1:**
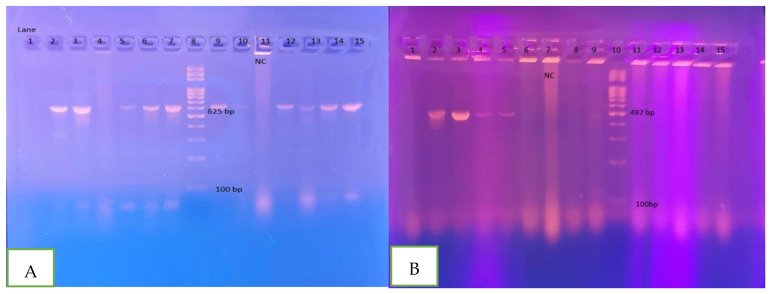
Agarose gel electrophoresis of DNA fragments generated by PCR for *fliC* gene (**A**) and *rfbE* gene (**B**) of *E. coli* O157:H7 isolates. In (**A**), Lane 8: DNA molecular weight marker (100 bp); Lane 11: Negative control; Lanes 2, 3, 5, 6, 7, 9, 10, 12, 13, 14, 15: *E. coli* O:157:H7 isolates. In (**B**), Lane 10: DNA molecular weight marker (100 bp); Lane 7: Negative control; Lanes 2, 3, 4, 5, 9: *E. coli* O:157:H7 isolates.

Suspected *E. coli* samples that formed colorless, non-lactose fermenting colonies on TC-SMAC agar, which is characteristic of *E. coli* O157:H7, were further analyzed using PCR with primers specific to the *rfbE* and *fliC* genes. An amplicon of 497 bp was considered indicative of the *rfbE* gene, while a 625 bp amplicon indicated the presence of the *fliC* gene, as illustrated in Figure 1. Out of the 250 *E. coli* isolates tested, 12 (4.8%) were confirmed as positive for the *E. coli* O157:H7 serotype.

### 3.2. Antimicrobial Susceptibility Test

All *E. coli* O157:H7 isolates were assessed for antibiotic susceptibility to the selected antimicrobial agents, with inhibition zones measured and categorized as resistant, intermediate, or sensitive. The highest resistance was observed against ampicillin, with a resistance rate of 91%. Resistance to trimethoprim/sulfamethoxazole, piperacillin, gentamicin, ciprofloxacin, ceftriaxone, and amoxicillin/clavulanic acid ranged from 51% to 75%. Conversely, high sensitivity rates were recorded for meropenem, imipenem, piperacillin/tazobactam, and aztreonam, as detailed in Table 2.

### 3.3. Broth Microdilution Testing Against Gentamicin

The MIC values for gentamicin ranged from 1 to 64 μg/mL. Among the *E. coli* O157:H7 isolates, eight demonstrated resistance to gentamicin, with an MIC of 64 μg/mL, while four isolates were classified as susceptible, exhibiting MICs of ≤2 μg/mL.

### 3.4. The Antimicrobial Activity of Commercial Probiotics Against E. coli O157:H7 Isolates

Two commercial probiotics were evaluated for their antimicrobial activity against *E. coli* O157:H7. Probiotic (P1) contained a single strain of *Saccharomyces boulardii*, while Probiotic (P2) consisted of a combination of *Saccharomyces boulardii*, *Bifidobacterium bifidum*, *Lactobacillus acidophilus*, and *Lactobacillus rhamnosus*. The antimicrobial effect was tested using the agar well diffusion method. However, the results demonstrated that neither probiotic inhibited the growth of *E. coli* O157:H7 isolates.

### 3.5. The Antibacterial Effect of Probiotics in Combination with Gentamicin

**Figure 2:**
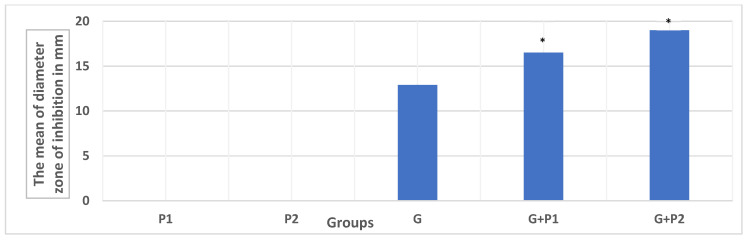
The antimicrobial effect of single and multi-strained probiotics alone and in combination with gentamicin against *E. coli* O157:H7 isolates. Values represent the mean inhibition zone (in millimeters) ± standard deviation, calculated from multiple replicates for each treatment. * The *p*-value of the mean diameter of the zone of inhibition of different groups was <0.05 using one-way ANOVA test followed by Tukey’s test, compared to gentamicin.

The current study demonstrated that the combination of gentamicin and probiotics produced significantly larger inhibition zones against *E. coli* O157:H7 compared to the use of gentamicin alone (*p* < 0.05). This effect was observed across all 12 *E. coli* O157:H7 isolates tested. The mean inhibition zone for gentamicin alone was 12.9 ± 2.27 mm. When combined with the single-strain probiotic P1, the inhibition zone increased to 16.5 ± 2.24 mm, and with the multi-strain probiotic P2, it further increased to 19 ± 2.8 mm. These results indicate that probiotics enhance the antimicrobial activity of gentamicin, with the multi-strain probiotic exhibiting a more pronounced potentiating effect (refer to Figure 2).

### 3.6. Assessment of Pathological Manifestations and Survival Outcomes in Mice Groups

**Figure 3:**
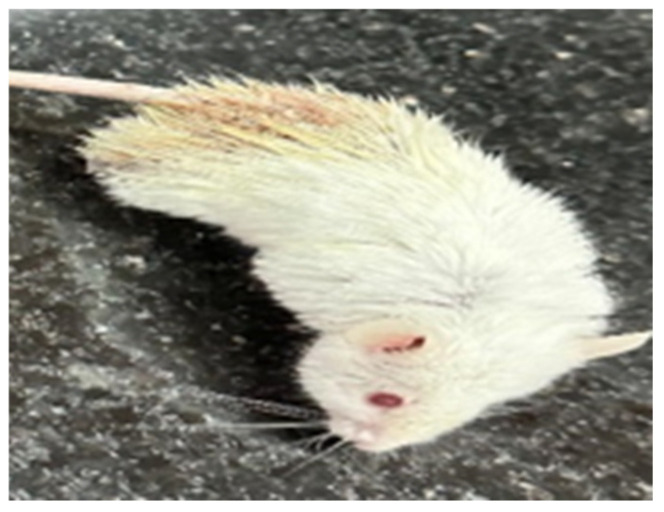
A picture of infected mice with bruising and fur abnormalities.

In this study, various groups of mice were assessed for pathological manifestations and survival rates following different treatments. The control group exhibited no signs of pathology and maintained a 100% survival rate, providing a baseline for comparison. The infected group, in contrast, showed severe pathological signs, including diarrhea, bruising, as seen in Figure 3, fur abnormalities, and decreased activity, with a high mortality rate (refer to Table 3). The P1 group demonstrated moderate adverse effects, with diarrhea present in all mice, and bruising and fur abnormalities in a subset, resulting in a survival rate of 71.4% (2/7 deaths). The P2 group showed no pathological symptoms and achieved a 100% survival rate, indicating its effectiveness in preventing infection-related pathology. Similarly, the gentamicin group (G) also displayed no pathological signs and had a perfect survival rate. In the G+P1 group, a survival rate of 85.7% was observed, with fewer symptoms than the infected or P1 groups. This group showed no diarrhea, but mild bruising and decreased activity were noted in some mice. Finally, the G+P2 group exhibited no pathological manifestations, with 100% survival, similar to the G and P2 groups, suggesting that this combination treatment was highly effective in preventing infection-induced health issues.

Over the course of the 7-day experiment, the weight changes in the various mouse groups were monitored to evaluate the effects of the different treatments. The control group exhibited consistent weight gains, with a final weight change of +4.7 ± 1.9 g, indicating healthy growth. In contrast, the infected group displayed significant weight loss, especially on days 2 and 3, followed by a gradual recovery, but ultimately showed a modest weight change of +0.3 ± 1.5 g by day 7 (*p* < 0.05 compared to the control group). The P1 treatment group also experienced early weight loss, but showed partial recovery by the end of the study, with a final weight change of +1.45 ± 1.7 g (*p* < 0.05 compared to the control group). The P2 group, however, demonstrated a steady positive weight change throughout the experiment, culminating in a final weight gain of +3.02 ± 1.3 g by day 7 (*p* < 0.05 compared to the infected group), indicating a strong potential for improving health and recovery during infection. The G treatment group followed a similar pattern, showing a final weight change of +3 ± 1.3 g (*p* < 0.05 compared to the infected group), further supporting the effectiveness of this treatment in maintaining weight and promoting recovery. The G+P1 group showed modest weight gain, with a final change of +2.12 ± 1.8 g (*p* < 0.05 compared to the control group), while the G+P2 group showed excellent recovery, with a final weight change of +2.6 ± 1.03 g (*p* < 0.05 compared to the infected group), suggesting a combined positive effect of G and P2. These findings, detailed in Table 4 demonstrate that treatments P2, G, and G+P2 promoted significant weight recovery compared to the infected group, which experienced notable weight loss.

### 3.7. Hematological and Biochemical Changes in Mice Groups

In this study, we assessed various hematological and biochemical parameters to evaluate the impact of different treatments on the mice, focusing on bacterial infection, hemolytic anemia, thrombocytopenia, and nephrotoxicity. The control group showed normal values across all parameters, indicating a healthy state. However, the infected group exhibited significant changes due to the bacterial infection. Hemolytic anemia was evident, with significant reductions in red blood cell (RBC) count (4.6 ± 0.3 × 10^6^/μL), hematocrit (HCT) (30 ± 2.1%), and hemoglobin (HG) (10.4 ± 0.9 g/dL). Thrombocytopenia was also observed, with a decrease in platelet (PLT) count (244 ± 44 × 10^3^/μL). Additionally, the white blood cell (WBC) count increased significantly (13.3 ± 3.02 × 10^3^/μL) alongside neutrophils (71.3 ± 5.1%), reflecting the immune response to the infection. Kidney function was impaired, as indicated by elevated creatinine levels (0.81 ± 0.12 mg/dL), suggesting renal stress (refer to Table 5).

All treatment groups demonstrated significant improvements in hematological parameters when compared to the infected group. This indicates that the treatments effectively alleviated the hemolytic anemia and thrombocytopenia induced by the infection. However, the key difference between the treatment groups was observed in kidney function, specifically in the creatinine levels.

P1 treatment showed some improvement in hematological parameters, but it resulted in worsened kidney function, with significantly higher creatinine levels (0.63 ± 0.15 mg/dL) compared to the control groups (*p* < 0.05), indicating that P1 may have limited effectiveness in improving renal function. In contrast, P2 treatment showed substantial improvements in both hematological and renal parameters. RBC (7.9 ± 0.5 × 10^6^/μL), HCT (39.7 ± 1.1%), and HG (13.7 ± 0.8 g/dL) levels were significantly restored, and PLT count (464.8 ± 292 × 10^3^/μL) showed recovery. Moreover, creatinine levels in the P2 group (0.24 ± 0.2 mg/dL) were significantly lower than those in the infected group (*p* < 0.05), suggesting that P2 was effective in reducing both the hematological disturbances and kidney dysfunction.

The gentamicin (G) group showed some improvement in hematological parameters. However, this group exhibited elevated creatinine levels (0.43 ± 0.09 mg/dL), indicating some nephrotoxic effect. Similarly, the G+P1 and G+P2 groups showed increased creatinine levels (0.53 ± 0.19 mg/dL and 0.5 ± 0.23 mg/dL, respectively), indicating potential kidney damage, despite improvements in hematological parameters.

### 3.8. Histological Parameters

**Figure 4:**
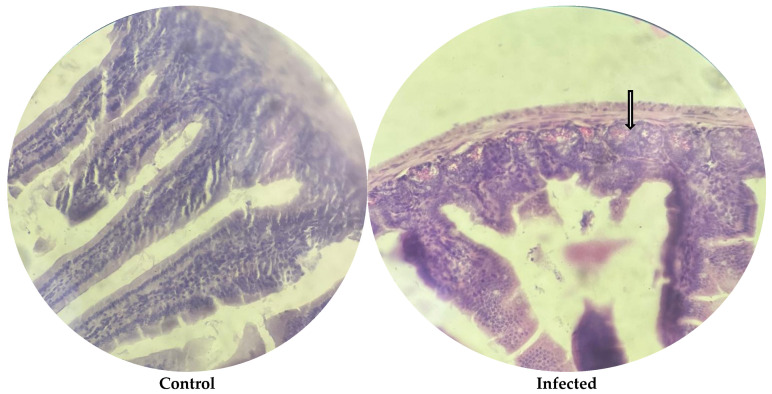
Histopathology in *E. coli* O157:H7-infected mice. Normal intestinal histology was observed in the control group. Infected mice exhibited atrophic villi and intraglandular bleeding (indicated by the arrow).

**Figure 5:**
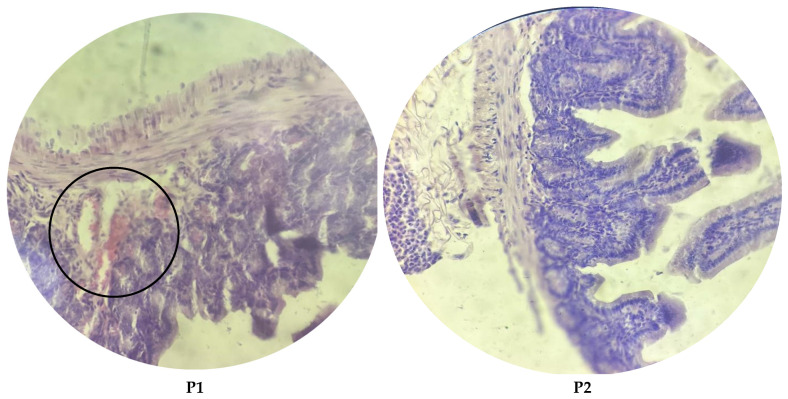
Histopathology in *E. coli* O157:H7-infected mice. Normal intestinal histology was observed in the P2 group. Mice treated with P1 displayed intraglandular bleeding (highlighted by circle).

**Figure 6:**
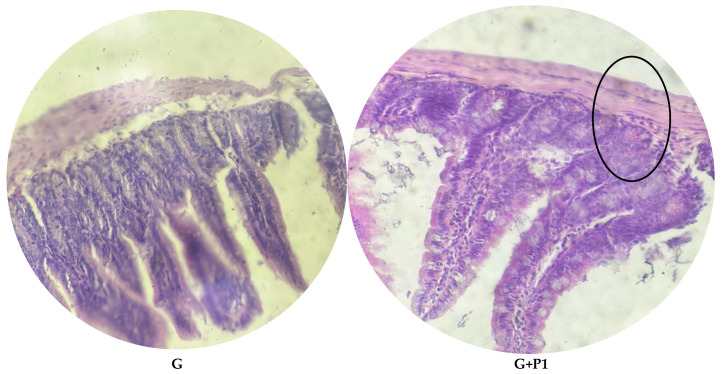

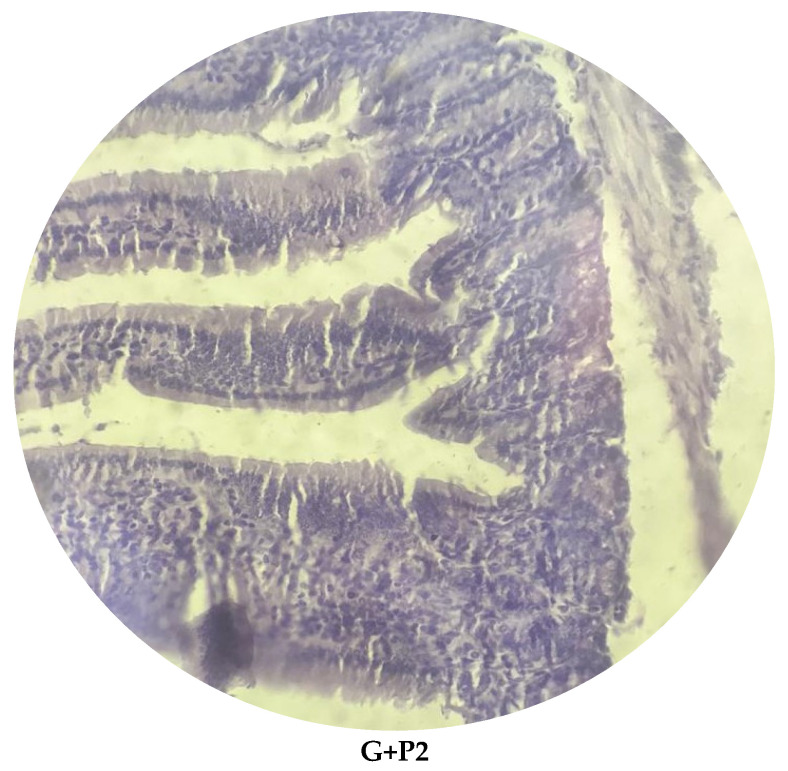
Histopathology in *E. coli* O157:H7-infected mice. Normal intestinal histology was observed in the gentamicin group and the G+P2 combination group. Mice in the G+P1 combination group exhibited mild intraglandular bleeding (highlighted by the circle).

Histological analysis of the ileal tissue revealed marked differences among the experimental groups, ranging from severe damage in the infected group to near-complete recovery in several treatment groups, as illustrated in Figure 4, Figure 5 and Figure 6. In the control group, the ileal structure, including the villi, mucosa, submucosa, and glands, remained intact and showed no abnormalities. In contrast, the infected group showed significant pathological changes characterized by villous atrophy and intraglandular bleeding, consistent with the harmful effects of *E. coli* O157:H7 infection. Treatment responses varied across groups. The P1 group demonstrated partial recovery, with reduced glandular bleeding, though some structural damage persisted. In the P2, G+P2, and G groups, tissue integrity was largely restored, with villi and glands displaying near-normal architecture. However, the G+P1 group showed residual intraglandular bleeding, indicating incomplete recovery despite treatment.

**Figure 7:**
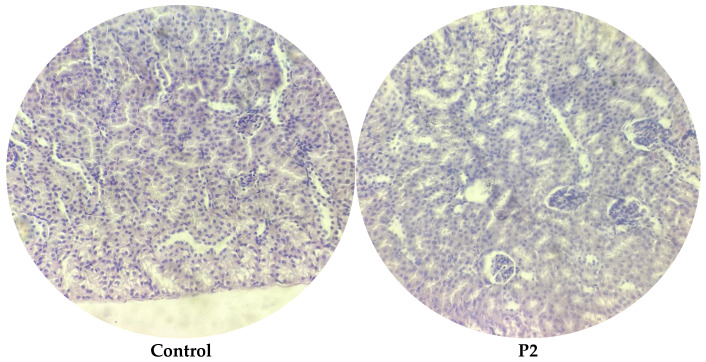
Renal histopathology in *E. coli* O157:H7-infected mice. Preservation of normal cortex in the control and P2 treatment group.

**Figure 8:**
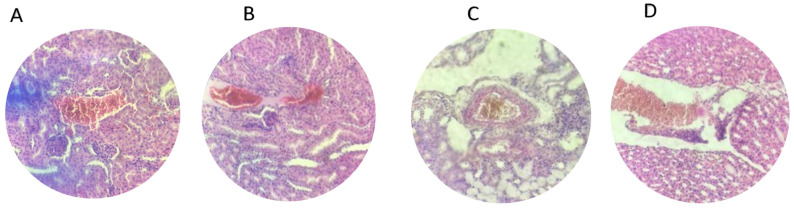
Renal histopathological changes in *E. coli* O157:H7-infected mice without treatment. Panel (**A**) shows bleeding in the cortical region, Panel (**B**) illustrates bleeding in the collecting duct, Panel (**C**) highlights congested blood vessels in the collecting duct, and Panel (**D**) displays bleeding in the calyceal system.

**Figure 9:**
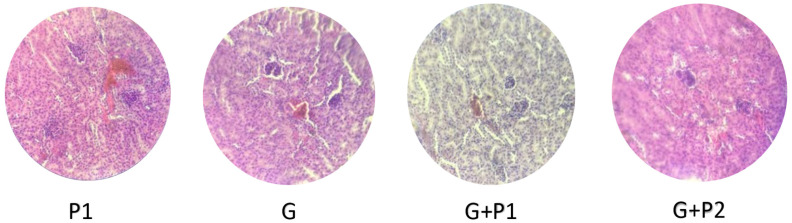
Renal histopathological changes in *E. coli* O157:H7-infected mice. Bleeding in the cortex was observed in all treatment groups, with the most severe bleeding in the P1 group and the least seen in mice treated with the gentamicin+P2 combination.

Similarly, the microscopic examination of renal tissues demonstrated clear differences between the experimental groups. In the control group, the cortex and collecting tubules maintained normal tissue architecture with no signs of pathology, as shown in Figure 7. In contrast, the infected group exhibited significant hemorrhaging in the collecting ducts, calyceal system, and cortex, consistent with the damage caused by *E. coli* O157:H7 infection (Figure 8). Mice treated with the single-strain probiotic (P1) displayed noticeable bleeding within the cortex, indicating partial improvement, although some damage remained. Contrarily, the P2-treated group showed complete recovery, with kidney tissues returning to their normal structure, demonstrating the effective restoration of renal integrity. In the gentamicin group, mild residual bleeding was observed in the cortex, suggesting limited remaining effects of the infection. Treatment with a combination of gentamicin and P1 (G+P1) resulted in minor bleeding in the collecting ducts, while the G+P2 group exhibited slight traces of bleeding in the cortex. Despite these mild residual effects, the G+P2 group showed significant restoration of kidney integrity (Figure 9).

## 4. Discussion

Over the past decade, probiotics have gained growing interest for their potential health benefits, particularly their antimicrobial effects and ability to address antibiotic-resistant pathogens [57,58,59]. Given the alarming antibiotic resistance rates observed in our study—where 91% of isolates were resistant to ampicillin and 66–75% showed resistance to trimethoprim/sulfamethoxazole and gentamicin—the need for such alternative treatments has become more urgent. In light of these concerning findings, our research aimed to evaluate the effectiveness of single-strain and multi-strain probiotics, both independently and in combination with gentamicin, through in vitro and in vivo experiments targeting *E. coli* O157:H7. Our results revealed that combining gentamicin with probiotics (G+P1, G+P2) led to both potentiating and synergistic effects in vitro, with the multi-strain probiotic P2 showing the greatest improvement in gentamicin’s effectiveness. These observations align with earlier research indicating that combining synthetic antibiotics with probiotics can significantly enhance bacterial inhibition or pathogen elimination, often producing larger inhibition zones than antibiotics used alone [29,53,60].

Following the promising in vitro results, the in vivo experiment evaluated the effectiveness of probiotics, both alone and in combination with gentamicin, in reducing the harmful effects of *E. coli* O157:H7 infection in a mouse model. The study focused on survival rates and overall health outcomes. Infected mice exhibited severe symptoms, including early weight loss and increased mortality. These findings align with those reported by Karpman et al. (1997) [54], although the neurological symptoms observed in their study were not present in our infected mice. The infected group treated with the multi-strain probiotic P2, gentamicin, and the combination of gentamicin and P2 showed complete survival, no visible symptoms, and significant weight recovery by day 7 post-infection. These treatments successfully prevented infection-related damage and facilitated recovery. In contrast, the mice treated with the single-strain probiotic P1 exhibited symptoms, reduced survival, and limited weight recovery, indicating that P1 was less effective than P2 and gentamicin. Although combining gentamicin with P1 improved survival and weight gain, the effect was not as pronounced as with P2 or the gentamicin+P2 combination. The greater effectiveness of the multi-strain probiotic P2 compared to the single-strain probiotic P1 is likely due to the synergistic interactions among the bacterial strains in P2, resulting in improved adhesion and stronger pathogen inhibition, as explained by MacFarland et al. Moreover, the inclusion of multiple strains offers a variety of mechanisms of action, ensuring broader coverage in restoring microbiome balance [61]. These findings align with those of other researchers, further supporting the enhanced efficacy of the multi-strain probiotic P2 over the single-strain P1, whether used alone or in combination with gentamicin [62,63,64,65,66,67].

Hematological and biochemical analyses showed that the infected group exhibited hemolytic anemia, thrombocytopenia, and elevated WBC and neutrophil counts. Increased creatinine and BUN levels were also observed, indicating infection-induced kidney dysfunction. These results are consistent with the typical infection caused by *E. coli* O157:H7. Similarly, Mohawk et al. and Keepers et al. reported elevated blood urea nitrogen levels and renal tubular damage in *E. coli* O157:H7-infected animals, further confirming our results [68,69]. Among the treatment groups, the multi-strain probiotic P2 exhibited the most substantial improvements in red blood cell count, hematocrit, and hemoglobin levels. It also effectively reduced creatinine levels, suggesting recovery in both hematological and renal functions. Gentamicin also improved blood parameters, but it caused higher creatinine levels, which is a known sign of kidney damage induced by gentamicin [70,71]. Huang and his colleagues also observed increased creatinine levels in mice treated with gentamicin [72]. This rise in creatinine was evident in all gentamicin combinations. The combination of gentamicin and multi-strain probiotic P2 showed better hematological outcomes, with a slight increase in creatinine levels. In contrast, the P1 group and the G+P1 combination were less effective with minimal improvements in kidney function. These results highlight the nephrotoxic effects of gentamicin and underline the superior efficacy of multi-strain probiotic P2 in vivo.

The histological evaluation further supported these findings by showing the tissue-specific effects of the different treatment groups. In the infected group, severe pathological changes were observed in the ileal and kidney tissues, including villous atrophy, intraglandular bleeding, and cortical hemorrhage. Similar to our findings, mouse models of *E. coli* O157:H7 infection also exhibited atrophic villi, hyperemia, and kidney pathological changes [73,74,75]. Treatment with the multi-strained probiotic P2 led to near-complete restoration of both ileal and kidney architecture, demonstrating its efficacy in reversing infection-induced damage. In contrast, the G and G+P2 groups showed significant tissue recovery with mild residual kidney damage. The P1 and G+P1 groups showed partial recovery, with ongoing tissue damage, particularly in the kidneys. These histological findings align with the hematological and biochemical results, therefore confirming the superior ability of the multi-strained probiotic P2 to promote tissue recovery and overall health compared to gentamicin and its combinations.

Interestingly, although multiple studies have demonstrated that probiotics can reduce the pathogenic effects of *E. coli* O157:H7 and inhibit its growth in vitro, our results revealed that neither single-strain nor multi-strain probiotics were able to inhibit the growth of *E. coli* O157:H7 in vitro [76,77,78]. In vivo experiments, however, produced more promising results, with multi-strain probiotics showing greater efficacy than single-strain probiotics. This discrepancy may be attributed to the different mechanisms of action of probiotics. Previous research suggests that probiotics combat enteric pathogens through three main mechanisms: direct antagonism, immunomodulation, and competitive exclusion [79,80,81,82]. In our study, we observed no evidence of direct antagonism, as the bacteria were not eradicated in vitro, suggesting that the benefits of probiotics may stem more from their immunomodulatory effects or their ability to reduce pathogen colonization rather than direct pathogen elimination, as described by Turkova et al. [83].

### Strengths and Limitations of the Study

This study has several key strengths. The use of both in vitro and in vivo models allowed for a comprehensive evaluation of probiotics against *E. coli* O157:H7, providing mechanistic insights and practical evidence of efficacy. The assessment of multiple outcomes, including survival rates, weight recovery, and tissue histology, highlighted the therapeutic potential of multi-strain probiotics and their synergistic effect when combined with gentamicin. These findings support the potential of probiotic-antibiotic combinations in enhancing treatment effectiveness and reducing infection-related complications. However, the study has limitations. The in vitro experiments do not fully imitate the host–pathogen interactions, and the mouse model may not perfectly represent human responses to *E. coli* O157:H7 infections. Gentamicin-induced kidney toxicity remains a concern, stressing the need for alternative antibiotics or optimized regimens. Additionally, the focus on specific commercial probiotic strains limits the applicability of the findings to other formulations.

## 5. Conclusions

This study emphasizes the therapeutic potential of combining multi-strain probiotics with gentamicin to address the pathological, physiological, and histological impacts of *E. coli* O157:H7 infection. The multi-strain probiotic consistently demonstrated superior efficacy, positioning it as a promising treatment option for *E. coli* O157:H7 infections. While gentamicin also showed effectiveness, its nephrotoxic effects must be carefully considered in clinical settings. The combination of multi-strain probiotic with gentamicin (G+P2) provided additional benefits but did not fully alleviate nephrotoxicity, highlighting the need for further optimization of combination therapies. Future research should focus on understanding the mechanisms behind the protective effects of multi-strain probiotics and evaluating their potential for broader use in managing *E. coli* O157:H7 infections.

## Figures and Tables

**Table 1:** Constituent strains of probiotics, as described by the manufacturer.

Single-Strain Probiotic (P1)	*Saccharomyces boulardii.*	5 × 10^9^ CFU/mL
Multi-Strain Probiotic (P2)	*Saccharomyces boulardii*	1 × 10^9^ CFU/mL
	*Bifidobacterium bifidum*	5 × 10^9^ CFU/mL
	*Lactobacillus acidophilus*	4 × 10^9^ CFU/mL
	*Lactobacillus rhamnosus*	4 × 10^9^ CFU/mL

**Table 2:** The antimicrobial susceptibility pattern of *E. coli* O157:H7 isolates (n = 12).

	Resistant	Intermediate	Sensitive
Piperacillin/tazobactam	-	1	11
Piperacillin	8 (66.6%)	3	1
Ampicillin	11 (91%)	-	1
Amoxicillin/clavulanic	5 (51%)	5	2
Ceftriaxone	7 (58%)	1	4
Cefepime	2 (16%)	4	6
Ceftazidime	1 (8%)	2	9
Ciprofloxacin	7 (58%)	5	-
Norfloxacin	4 (33%)	2	6
Imipenem	-	2	10
Meropenem	-	-	12
Aztreonam	3 (25%)	1	8
Gentamicin	8 (66.6%)	-	4
Trimethoprim/sulfamethoxazol	9 (75%)	-	3
Chloramphenicol	3 (25%)	2	7

**Table 3:** Pathological manifestations and survival rate observed in mice groups during the course of the experiment.

	Number of Deaths	Survival Rate	Diarrhea	Bruising	Flurred Fur	Decreased Activity
Control	0/7	100%	−	0/7	0/7	0
Infected	4/7	42.8%	+	4/7	4/7	4/7
P1	2/7	71.4%	+	1/7	3/7	1/7
P2	0/7	100%	−	0/7	0/7	0/7
G	0/7	100.00%	−	0/7	0/7	0/7
G+P1	1/7	85.70%	−	1/7	0/7	1/7
G+P2	0/7	100%	−	0/7	0/7	0/7

**Table 4:** The weight changes in mice in different groups over 7 days post-infection.

	dd2	dd3	dd4	dd5	dd6	dd7
Control	1.16 ± 1.2	1.34 ± 1.5	2.19 ± 1.4	3.12 ± 0.9	3.57 ± 1.4	4.7 ± 1.9
Infected	−0.66 ± 1.3	−0.3 ± 1.4	0.24 ± 1.2 *	0.163 ± 1.4 *	−0.5 ± 1.9 *	0.3 ± 1.5 *
P1	0.4 ± 1.1	0.0625 ± 1.7	1.28 ± 1.7	1.6 ± 1.4	2.05 ± 1.5	1.45 ± 1.7 *
P2	0.57 ± 1.41	0.76 ± 1.01	1.3 ± 0.7	1.78 ± 0.6	2.42 ± 1.05 **	3.02 ± 1.3 **
G	0.35 ± 1.7	0.26 ± 1.03	1.4 ± 1.1	2.08 ± 1.3 **	2.6 ± 1.5 **	3 ± 1.3 **
G+P1	0.25 ± 1.2	0.25 ± 1.3	1.01 ± 1.4	1.68 ± 1.6	1.88 ± 1.9	2.12 ± 1.8 *
G+P2	0.3 ± 1.8	1.43 ± 1.9	1.41 ± 1.7	2.25 ± 1.7 **	3.16 ± 0.9 **	2.6 ± 1.03 **

The values presented in this table represent the mean weight change ± standard deviation (SD). Daily weight change was calculated by subtracting the initial weight (prior to bacterial inoculation) from the subsequent weight. Statistical analysis of mean weight changes was performed using one-way analysis of variance (ANOVA). * indicates that the mean values were significantly different from the control group (*p* < 0.05). ** indicates that the mean values were significantly different from the infected group (*p* < 0.05).

**Table 5:** Hematological and biochemical changes in mice groups.

	WBC ×10^3^/μL	RBC ×10^6^/μL	HCT %	HG g/dL	PLT ×10^3^/μL	Neutrophils %	BUN mg/dL	Cr mg/dL
Control	4.46 ± 1.04	8.2 ± 0.3	40 ± 1.7	13.7 ± 0.9	483 ± 85	32.5 ± 2.7	18.4 ± 2.3	0.24 ± 0.02
Infected	13.3 ± 3.02 *	4.6 ± 0.3 *	30 ± 2.1 *	10.4 ± 0.9 *	244 ± 44	71.3 ± 5.1 *	24.3 ± 4.2	0.81 ± 0.12 *
P1	4.69 ± 1.7 **	7.08 ± 1.1 **	36.7 ± 1.4 **	12.9 ± 1.6 **	303.4 ± 138	17.14 ± 2.6 **	17 ± 2.16 **	0.63 ± 0.15 * €
P2	5.6 ± 1.11 **	7.9 ± 0.5 **	39.7 ± 1.1 **	13.7 ± 0.8 **	464.8 ± 292	20.6 ± 2.6 **	14.8 ± 1.4 **	0.24 ± 0.2 **
G	4.7 ± 1.7 **	6.2 ± 0.9 **	35.9 ± 1.03 **	12.2 ± 1.16 **	243.25 ± 56	27.1 ± 6 **	15.6 ± 5.1 **	0.43 ± 0.09 **
G+P1	4.89 ± 1.4 **	7.4 ± 0.8 **	38.7 ± 2.3 **	13.3 ± 0.8 **	372.5 ± 177	19.4 ± 5.2 **	19.8 ± 3.2	0.53 ± 0.19 * ** €
G+P2	4.49 ± 1.4 **	7.03 ± 0.57 **	39 ± 2 **	13 ± 0.69 **	381.4 ± 161	17.1 ± 3.3 **	17.2 ± 2.3 **	0.5 ± 0.23 * ** €

The values presented in this table represent the mean weight change ± standard deviation (SD). Statistical analysis of mean weight changes was performed using one-way analysis of variance (ANOVA) followed by Tukey test. * indicates that the mean values were significantly different from the control group (*p* < 0.05). ** indicates that the mean values were significantly different from the infected group (*p* < 0.05). € indicates that the mean values were significantly different from the P2 group (*p* < 0.05).

## Data Availability

The raw data supporting the conclusions of this article will be made available by the authors on request.
